# HDL endocytosis and resecretion^☆^

**DOI:** 10.1016/j.bbalip.2013.07.014

**Published:** 2013-11

**Authors:** Clemens Röhrl, Herbert Stangl

**Affiliations:** Department of Medical Chemistry, Center for Pathobiochemistry and Genetics, Medical University of Vienna, Vienna, Austria

**Keywords:** Lipoprotein, Holo-particle uptake, Cholesterol, Transcytosis, Resecretion, Degradation

## Abstract

HDL removes excess cholesterol from peripheral tissues and delivers it to the liver and steroidogenic tissues via selective lipid uptake without catabolism of the HDL particle itself. In addition, endocytosis of HDL holo-particles has been debated for nearly 40 years. However, neither the connection between HDL endocytosis and selective lipid uptake, nor the physiological relevance of HDL uptake has been delineated clearly. This review will focus on HDL endocytosis and resecretion and its relation to cholesterol transfer. We will discuss the role of HDL endocytosis in maintaining cholesterol homeostasis in tissues and cell types involved in atherosclerosis, focusing on liver, macrophages and endothelium. We will critically summarize the current knowledge on the receptors mediating HDL endocytosis including SR-BI, F_1_-ATPase and CD36 and on intracellular HDL transport routes. Dependent on the tissue, HDL is either resecreted (retro-endocytosis) or degraded after endocytosis. Finally, findings on HDL transcytosis across the endothelial barrier will be summarized. We suggest that HDL endocytosis and resecretion is a rather redundant pathway under physiologic conditions. In case of disturbed lipid metabolism, however, HDL retro-endocytosis represents an alternative pathway that enables tissues to maintain cellular cholesterol homeostasis.

## Highlights

• HDL endocytosis and resecretion is accompanied by cholesterol exchange.
• HDL endocytosis and resecretion might be an alternative pathway for lipid exchange.
• HDL is catabolized by the liver and in tissues with high cholesterol demand.
• Several redundant receptors were shown to mediate HDL endocytosis.
• HDL endocytosis might be relevant in hepatocytes, endothelial cells and macrophages.

## 1 Introduction

High-density lipoprotein (HDL) exerts multiple actions on metabolic homeostasis and thereby beneficially impacts atherosclerosis, thrombosis, inflammation, and glucose homeostasis. Importantly, HDL removes excess cholesterol from peripheral tissues and cells. These cells include macrophage foam cells, which are directly involved in atherosclerotic plaque formation. HDL then transports this cholesterol to the liver for disposal into the bile. This process of peripheral cholesterol efflux to HDL and delivery to the liver is termed reverse cholesterol transport (RCT) and is important for the anti-atherogenic properties of HDL. Thus, high plasma HDL cholesterol levels are generally associated with reduced risk for cardiovascular diseases [1,2].

Recent studies suggest that HDL is not a mere transport vehicle for lipids, but is also a carrier of non-lipid cargo such as microRNAs [3]. Further, HDL can influence cellular signaling by binding to its cell surface receptors. Hence, the complexity of HDL metabolism is still incompletely understood, which also holds true for our knowledge on the transfer of lipids between HDL and target cells. This is in contrast to our extensive knowledge on the metabolism of low-density lipoprotein (LDL): LDL bound to the LDL receptor (LDL-R) is internalized via clathrin-coated pits, which fuse with early sorting endosomes. After dissociation from its receptor, the majority of LDL particles are delivered to late endosomal compartments for hydrolysis of lipids and protein degradation [4]. Free cholesterol can then exit late endosomes towards the endoplasmic reticulum or the plasma membrane. HDL metabolism, however, is more complex with regard to the cholesterol transport routes and HDL binds to a variety of cells with varying degrees of specificity. Accordingly, a number of proteins and receptors have been described to bind HDL. After receptor binding, HDL transfers lipids to cells predominantly by selective lipid uptake without catabolism of the particle [5]. In addition, an alternative pathway comprising endocytosis of whole HDL particles followed by resecretion was observed more than 40 years ago. However, neither the connection between HDL endocytosis and selective lipid uptake, nor the physiological relevance of HDL uptake is fully clarified.

In this review, we will summarize receptors and mechanisms for HDL endocytosis and resecretion focusing on liver, endothelial cells and macrophages as central tissues in atherosclerosis development.

## 2 HDL endocytosis and resecretion

Even though HDL endocytosis was described in the mid-seventies of the last century [6], the relevance of HDL endocytosis for HDL metabolism is still under debate and awaits its complete elucidation. Detailed analysis of HDL endocytosis was first described by Bierman, Stein and Stein in rat aortic smooth muscle cells. The authors suggested that HDL catabolism is low. Instead, they reported regurgitation of non-catabolized HDL by reverse endocytosis [6,7]. Next, HDL uptake and resecretion was described in macrophages by two independent studies of Alam et al. and Schmitz et al. [8,9]. In 1990 DeLamatre and co-workers demonstrated retro-endocytosis of iodinated HDL particles in a rat liver cell line [10]. However, the receptors involved in HDL binding were not identified at this time.

It was not until 1996 that scavenger receptor class B, type I (SR-BI) was cloned and characterized as an HDL receptor by the group of Krieger [11,12]. Its human ortholog CLA-1 was described by Calvo and Vega [13]. The main function of SR-BI is selective cholesteryl ester uptake of HDL derived lipids by the liver and steroidogenic tissues [14]. Indeed, SR-BI plays a crucial role in cholesterol homeostasis and reverse cholesterol transport: Hepatic SR-BI over-expression increases clearance of plasma cholesterol in mice [15–17] and reduces atherosclerosis [18,19]. In contrast, loss of hepatic SR-BI results in elevated plasma cholesterol levels leading to atherosclerosis [20]. In humans, a low abundance mutation in SR-BI leads to increased plasma cholesterol and impaired steroid hormone synthesis, further underlining the importance of this receptor in lipid metabolism [21].

The transport of lipids from HDL to SR-BI is generally thought to occur via selective cholesteryl ester uptake, where only the lipid load of HDL is transferred without concomitant catabolism of the HDL particle itself [5,22–24]. In addition, SR-BI is a bona fide endocytic receptor as it mediates the internalization of lipopolysaccharides and facilitates hepatitis C virus entry [25–27]. Therefore, it is well conceivable that SR-BI also mediates HDL endocytosis. This connection was first described by the group of Tall: Their experiments in polarized hepatocytes and kidney cells showed that SR-BI mediates HDL transport to the endosomal recycling compartment and apical membrane regions [28]. Studies in our lab showed concomitant endocytosis of SR-BI and HDL which was followed by particle resecretion. HDL resecretion could be linked to cholesterol efflux, since resecreted HDL particles were enriched in cellular cholesterol [29].

Endocytosis and resecretion is not limited to HDL, but instead occurs with almost all lipoprotein classes: Similar to HDL endocytosis and resecretion, uptake and resecretion was described for LDL as well as Lp(a). It is estimated that approximately 20% of endocytosed LDL escapes lysosomal degradation [30–32]. Additionally, recycling of apoE is well described [for review: [33]]. ApoE derived from LDL and triglyceride-rich particles are resistant to degradation and are resecreted. A functional role for apoE recycling is implicated in the generation of apoE-rich HDL particles as well as cholesterol efflux [34–36].

By now, HDL endocytosis has been shown to occur in cell lines originating from various tissues (summarized in Table 1). In addition, HDL endocytosis has been observed in a variety of species including humans, rodents and even insects and crustaceans [37]. This suggests that HDL endocytosis is a virtually ubiquitous process. This excludes erythrocytes, where cholesterol exchange with lipoproteins is considered a passive process without participation of lipoprotein receptors [38,39].

## 3 Intracellular HDL trafficking and lipid exchange

While LDL endocytosis is known to occur via clathrin-coated pits [40], it is under debate if HDL endocytosis is a clathrin-dependent or independent process. Inhibition of clathrin-mediated endocytosis inhibited LDL internalization, but not HDL uptake suggesting clathrin-independent mechanisms in hepatocytes and SR-BI over-expressing CHO cells [28]. Caveolae are specialized bulb-shaped surface pits enriched in cholesterol containing caveolin and cavin proteins and are implicated in a variety of physiological processes including cell signaling and endocytosis. Interestingly, caveolin deficiency severely affects lipid homeostasis [41]. SR-BI was reported to preferentially localize to caveolae and not clathrin-coated pits in CHO cells over-expressing SR-BI [42]. In contrast, SR-BI is localized in rafts devoid of caveolin-1 in HepG2 cells [43]. In line with this observation, HDL is efficiently endocytosed via clathrin-coated pits in HepG2 cells, a cell line devoid of plasma membrane caveolae [44,45]. HDL endocytosis is also clathrin-mediated in Caco-2 cells [46]. Indeed, the caveolar density remarkably differs in different tissues. Caveolae are merely detectable in the kidney, whereas they make up to 50% of the plasma membrane surface in endothelial cells and adipocytes [41]. As HDL endocytosis seems to be a ubiquitous process, we suggest that caveolae are no pre-requisite for HDL endocytosis, although the presence of caveolae might account for a favorable environment to enhance HDL endocytosis.

**Fig. 1:**
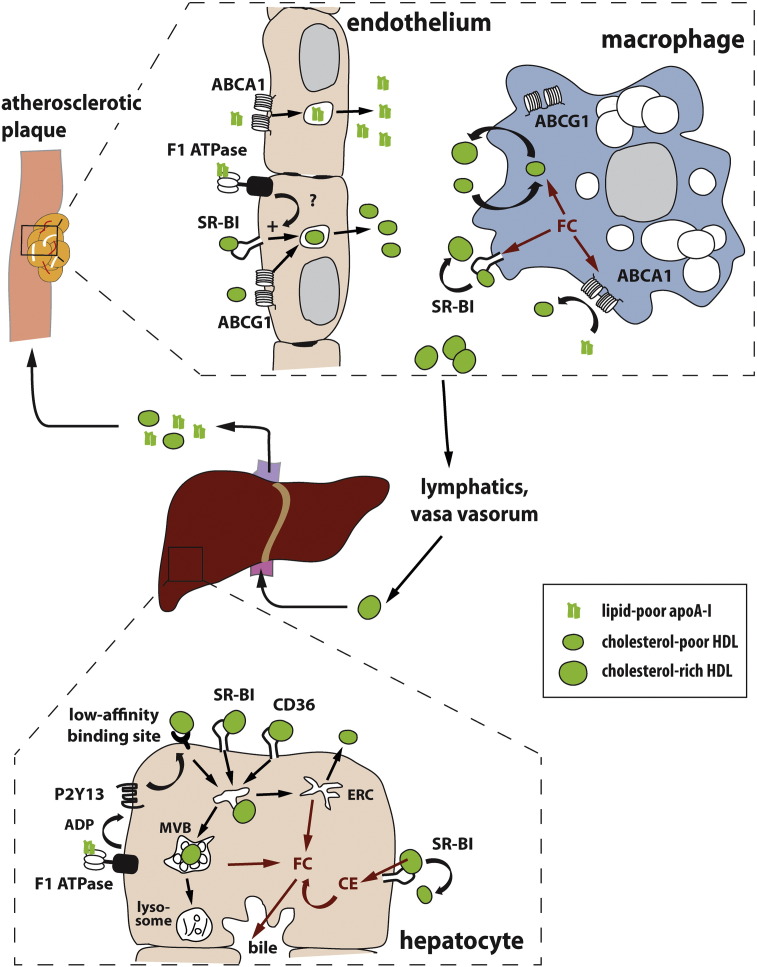
Putative contributions of HDL endocytosis and resecretion to RCT. ApoA-I is secreted by the liver and intestine (not shown here) and acquires phospholipids and cholesterol. To exert athero-protective effects, apoA-I and HDL have to be transported to macrophage foam cells through endothelial cells. ABCA1 is necessary for apoA-I transcytosis through endothelial cells, whereas SR-BI, ABCG1 and ecto-F_1_-ATPase facilitate HDL transport. Excess macrophage free cholesterol is transported to apoA-I by ABCA1 or to HDL by SR-BI. In addition, HDL retro-endocytosis was shown to mediate cholesterol efflux. ABCG1 mainly seems to have a role in intracellular cholesterol trafficking. Cholesterol enriched HDL then leaves the plaque via the lymphatics and the vasa vasorum and is transported back to the liver. Here, SR-BI transfers cholesterol to hepatocytes by selective lipid uptake. In addition, SR-BI, CD36 and a low affinity HDL binding site under the control of ecto-F_1_-ATPase and P2Y13 are discussed to mediate HDL holo-particle uptake. After endocytosis, HDL is either rapidly recycled through the endosomal recycling compartment (ERC) and resecreted or transported to multivesicular bodies (MVBs). HDL degradation in lysosomes is limited and occurs rather slowly. During endocytosis, HDL exchanges cholesterol with hepatocytes. Cholesterol is then either used for the formation of new lipoproteins or secreted into the bile directly or indirectly after conversion to bile-acids. Abbreviations: FC (free cholesterol); CE (esterified cholesterol); apoA-I (apolipoprotein A-I); HDL (high-density lipoprotein); SR-BI (scavenger receptor class B, type I); ABCA1 (ATP-binding cassette transporter A1); ABCG1 (ATP-binding cassette transporter G1); CD36 (cluster of differentiation 36).

Studies in our lab have focused on extensive characterization of HDL endocytosis using different methodologies: Using ultrasensitive fluorescence microcopy in SR-BI over-expressing cells we found that HDL is endocytosed in clusters of approximately 10 particles. HDL clusters are rapidly transported towards the Golgi-apparatus (v = 0.52 μm/s), a process requiring functional microtubules [47]. By means of combined light and electron microscopy, we further showed a central role of multivesicular bodies – intermediate organelles between early and late endosomes – in HDL endocytosis in HepG2 cells. HDL accumulated in multivesicular bodies rather slowly with a peak labeling after three hours. Further studies suggest that HDL taken up by HepG2 and COS-7 cells is transported to two intracellular pools: A rapid-turnover retroendocytotic pool (half-life: 3.8 min) and a slow-turnover pool that is eventually further transported to lysosomes for degradation [48]. These results are in close agreement with studies by Wüstner and colleagues, who likewise described a rapid turnover HDL recycling pool (half-life: 6.9 min) in polarized hepatocytes which likely represents the endosomal recycling compartment [49,50]. HDL transport to the ERC is consistent with findings by Silver et al. [28]. In addition to this rapid-turnover ERC pool, we propose that multivesicular bodies represent the slow-turnover pool for HDL endocytosis (Fig. 1) [44].

Although the intracellular trafficking during HDL endocytosis has been characterized in many aspects, it is still unclear, if endocytosis is definitely necessary for selective lipid uptake. Several lines of evidence suggest that HDL endocytosis is not required for selective uptake: Liposomes containing purified SR-BI are sufficient for selective CE uptake [51]. Moreover, blocking of HDL endocytosis by different experimental approaches does not interfere with selective uptake [52,53]. Further, HDL is not endocytosed by luteinized ovaries which require high amounts of cholesterol for steroid hormone synthesis [54]. In contrast, HDL recycling through the ERC was found to determine selective cholesterol exchange between HDL and hepatocytes [28,55]. Interestingly, SR-BII, a splicing variant of SR-BI, was described to mediate endocytosis of HDL. SR-BII over-expression was more efficient in enhancing HDL uptake, whereas over-expression of SR-BI was more efficient in increasing selective uptake [56]. This again suggests that selective lipid uptake is distinct from HDL endocytosis. However, differences between murine and human SR-BI have been reported: Selective uptake mediated by expression of CLA-1, the human analog of SR-BI, relies on endocytosis, whereas selective uptake mediated by murine SR-BI does not [57].

It is thus unclear if HDL endocytosis is essentially required for selective uptake. However, it is clear that cholesterol exchange occurs during endocytosis, as several reports suggest modification of the HDL particle after endocytosis: In SR-BI overexpressing cells HDL size is more heterogeneous after resecretion, indicating HDL particle modification during retro-endocytosis [29]. In hepatocytes, resecreted HDL was depleted in free and esterified cholesterol which suggests selective lipid uptake during endocytosis [55]. In contrast, HDL resecretion from cholesterol-laden macrophages results in secretion of enlarged, cholesterol enriched HDL [9]. Thus, the cellular cholesterol status might determine if HDL is cholesterol loaded or depleted during retro-endocytosis.

## 4 HDL catabolism

While the majority of LDL is catabolized and degraded in lysosomes, degradation of HDL is generally hard to quantify. In human fibroblasts, degradation of HDL less than 5% compared to LDL [58] and measurable HDL degradation is limited to certain cell types. Among these, placenta trophoblast cells are an interesting example displaying considerable HDL degradation. Trophoblasts represent the contact zone between maternal and fetal tissue and their cholesterol handling is especially interesting as the growing embryo most likely depends on the supply of maternal cholesterol, primarily in the first trimester [59]. In first trimester trophoblasts, both HDL degradation and selective uptake from HDL are high. In term trophoblasts, HDL degradation and selective uptake are decreased, but still elevated compared to other tissues [60]. Trophoblasts are thus capable of dismantling large amounts of HDL, probably to meet the high cholesterol requirements of the growing embryo. Our investigations provide evidence for a similar HDL degrading mechanism in a subclone of CHO7 cells, which was adapted to grow under low cholesterol conditions. We described that HDL degradation in both CHO7 and placenta BeWo cells was only partially blocked by chloroquine, indicating a mechanism distinct from lysosomal degradation, which is typical for LDL catabolism [61]. Instead, HDL degradation involved lipases and proteoglycans and probably did not occur intracellularly. Indeed, a role in HDL catabolism has been attributed to lipases, as both endothelial lipase and hepatic lipase contribute to HDL remodeling, which alters the clearance from the circulation by liver and kidney [62,63].

The liver as a central organ in lipoprotein metabolism does not only mediate selective CE uptake from HDL, but also HDL endocytosis and degradation. HDL degradation in hepatocytes seems to be regulated by leptin and is impaired in ob/ob mice [55]. Hepatic HDL catabolism is not mediated by SR-BI, as SR-BI-deficient mice show no defect in HDL catabolism [64,65]. Further, SR-BI-mediated retro-endocytosis in murine hepatocytes was independent of HDL degradation [28], which appears to be mediated by a different receptor that remains to be identified. The β-chain of ATP synthase (ecto-F_1_-ATPase; see below), is a possible candidate mechanism facilitating HDL degradation. The independence of HDL degradation of SR-BI makes inhibition of HDL degradation a putative therapeutic strategy for raising HDL levels without concomitant inhibition of cholesterol clearance from plasma by selective uptake.

In contrast to the liver, the kidney is generally not regarded as a central organ in lipoprotein metabolism, because lipoproteins are too large to pass the glomerular filtration barrier. However, an important role is attributed to the kidney in apoA-I catabolism. The role of the kidney in lipid metabolism as well as the function of the HDL receptors cubilin and megalin are excellently reviewed elsewhere [66].

Taken together, HDL degradation occurs in metabolically highly active tissues and in cases, where extracellular cholesterol is limited. It would be interesting to study HDL degradation in malignant tumors, as they might rely on high amounts of exogenous cholesterol for proliferation. Indeed, SR-BI expression correlates with the degree of malignancy in melanoma cells (Mikula, Röhrl and Stangl, unpublished data), suggesting an increased demand for cholesterol in malignant metastatic tumors.

## 5 HDL endocytosis in tissues relevant to atherosclerosis

### 5.1 Liver

The liver is a complex organ composed of various cell types of parenchymal (hepatocytes) and non-parenchymal (Kupffer cells, endothelial cells, stellate cells) origin. Besides nascent HDL formation, the liver plays a major role in the clearance of HDL derived cholesterol. After injection of radiolabeled HDL, 22% of liver associated HDL was associated with Kupffer and endothelial cells, suggesting the importance of these cell types for HDL metabolism [67]. In hepatocytes, HDL-cholesterol can be secreted into the bile, converted to bile acids, or secreted via newly synthesized lipoproteins. It has been suggested that HDL and not LDL is the primary source of bile-cholesterol in humans [68]. Consistent with the important role of HDL in reverse cholesterol transport – i.e. the transport of macrophage cholesterol to the feces via biliary excretion – hepatic SR-BI over-expression increases macrophage reverse cholesterol transport [69]. SR-BI is expressed at both the basolateral (sinusoidal) and the apical (bile-canalicular) membrane in murine liver [15]. In WIF-B cells, a model for polarized hepatocytes, SR-BI distribution between the basolateral and apical membrane is regulated by the cellular cholesterol status [70]. This polarized distribution is consistent with SR-BI being a receptor for basolateral to apical cholesterol transport into the bile. Whether SR-BI mediates biliary cholesterol secretion via holo-particle uptake, however, is under debate. It was shown that SR-BI mediates HDL endocytosis in polarized HepG2 cells. However, free cholesterol rapidly dissociated from HDL and was transported to the apical bile-canalicular membrane independent of HDL [50]. In contrast, Bodipy-CE as a marker for esterified cholesterol was transported to the bile-canalicular membrane concomitantly with HDL in polarized primary hepatocytes [28]. These conflicting results can be rationalized by different cellular trafficking of free and esterified cholesterol [71]. Probably intracellular HDL trafficking is not important for amphiphilic free cholesterol transport to bile-canaliculi, because free cholesterol rapidly dissociates from the HDL particle and is efficiently transported by non-vesicular mechanisms.

In contrast to these in-vitro studies in SR-BI over-expressing cells, SR-BI knockout mice display unchanged holo-HDL uptake into the liver, whereas selective lipid uptake is considerably diminished [72]. This argues against a role for SR-BI in hepatic HDL uptake. In line with a minor role of SR-BI in hepatic HDL endocytosis is the extensive work of Laurent Martinez on an SR-BI-independent mechanism of HDL internalization in hepatic cells in-vitro and in-vivo: This mechanism requires apoA-I binding to a high affinity receptor identified as the cell surface expressed β-chain of ATP synthase (ecto-F_1_-ATPase), which is normally localized in the inner mitochondrial membrane [73]. ApoA-I binding to ecto-F_1_-ATPase induces the hydrolysis of ATP to ADP which in turn activates HDL endocytosis by a low affinity receptor that remains to be identified (Fig. 1). This endocytosis is clathrin-mediated and dependents on the activation of the purinergic receptor P2Y_13_ by the generated ADP [74]. Indeed, HDL endocytosis was found to be dependent on the extracellular ADP concentration and on enzymes modulating these levels [75]. Further characterization of this pathway showed its dependence on the GTPase signaling as well as cytoskeletal reorganization [76]. The regulation of hepatic HDL endocytosis by P2Y_13_ was also confirmed in-vivo: P2Y_13_-deficient mice exhibited decreased hepatic HDL and cholesterol uptake as well as decreased bilary cholesterol secretion and total macrophage-to-feces RCT [77]. Thus, P2Y_13_ is putative therapeutic target as a regulator of hepatic HDL endocytosis [78]. Besides the liver, ecto-F_1_-ATPase plays a role in HDL transcytosis in endothelial cells (see below).

Another receptor for hepatic holo-HDL particle uptake is scavenger receptor cluster of differentiation 36 (CD36), as recently reported by Rinninger's group [79]. CD36 shows a high degree of sequence homology in its extracellular domain compared to SR-BI. CD36 is a high affinity HDL receptor; in addition, CD36 has a broad spectrum of ligand as it is know from SR-BI. Given the high homology it is not surprising that CD36 also mediates selective lipid uptake from HDL, although less efficiently than SR-BI [80,81]. Moreover, CD36 promotes HDL endocytosis, especially in the liver [79]. HDL uptake by CD36 was shown in both hepatocytes and non-parenchymal liver cells. Interestingly, non-parenchymal liver cells showed higher HDL holo-particle uptake than hepatocytes when normalized to cell protein.

### 5.2 Macrophages

Macrophages play a crucial role in the pathogenesis of atherosclerosis. Endothelial inflammation leads to recruitment and invasion of monocytes/macrophages which excessively internalize modified lipoproteins. Excess cholesterol uptake triggers macrophage foam cell formation and pronounced inflammatory responses. Subsequent accumulation of cells, lipid and matrix leads to necrotic breakdown of some lesions and thrombosis [82]. Nascent and mature HDL particles can accept cholesterol from macrophages in atherosclerotic lesions and thereby counteract foam cell formation (Fig. 1). Major cholesterol efflux pathways include aqueous diffusion as well as ABCA1, ABCG1 and SR-BI-dependent processes [83]. If HDL endocytosis and resecretion also contribute to macrophage cholesterol homeostasis, remains to be clarified (this question is also addressed by a review article by Cavelier and colleagues [84]).

HDL uptake and resecretion in cholesterol-laden mouse peritoneal macrophages was the topic of one of the first reports of HDL retro-endocytosis [8]. Subsequent to receptor-mediated binding, HDL was internalized and transported into endosomes as demonstrated using gold-labeled HDL. HDL containing endosomes did not fuse with lysosomes and consistently, degradation was limited. Instead, HDL containing compartments were found to interact with lipid droplets, which might lead to direct exchange of cholesterol from lipid droplets to HDL. In this study, HDL_2_ showed a higher uptake than HDL_3_ [8]. In a subsequent report, Alam and colleagues confirmed enhanced internalization of apoE-free HDL in human monocyte-derived macrophages upon cholesterol loading. They found internalized HDL to be resecreted as larger ApoE-containing HDL_2_-like particles and hypothesized that HDL resecretion facilitates removal of excess cholesterol from cells [9]. More recently, we have also described HDL endocytosis and resecretion in THP-1 monocytes/macrophages [29]. In contrast, two studies report that HDL is not taken up in mouse peritoneal or RAW macrophages [85,86]. Although it is still unclear, if HDL retro-endocytosis contributes to cholesterol efflux, retro-endocytosis of free apoA-I might be important for cholesterol efflux [86,87]. Candidate receptors for HDL endocytosis in macrophages are the scavenger receptors SR-BI, CD36 and SR-A, which bind a broad spectrum of ligands as well as ABCG1, although the presence of this receptor at the plasma membrane has been questioned recently [88].

### 5.3 Endothelium

Interactions of lipoproteins with the vascular endothelium play a critical role in lipid homeostasis and atherosclerosis: First, endothelial cells mediate lipolysis of triglyceride-rich lipoproteins. Second, vascular tone and endothelial integrity can be regulated by lipoproteins [for review: [89]]. Third, lipoproteins as well as monocyte/macrophages have to pass the endothelium to interact with atherosclerotic lesions. HDL can pass the endothelial barrier more easily than VLDL and LDL, presumably due to its smaller size [90]. HDL is considered to be actively transported through endothelial cells by transcytosis, which involves apical endocytosis, intracellular trafficking and basolateral exocytosis. In arteries, HDL transcytosis only occurs from the plasma towards the intima and not in the reverse direction. Instead, HDL leaves the intima via the vasa vasorum and the lymphatics [91]. Recently, the important role of the lymphatics in removal of HDL from plaques and RCT was directly demonstrated [92].

Extensive work in the group of von Eckardstein revealed that both SR-BI and ABCG1 mediate HDL transcytosis in polarized bovine aortic endothelial cells (Fig. 1) [93]. This group showed a role for ecto-F_1_-ATPase in regulating HDL transcytosis [94]. If ecto-F_1_-ATPase is causally involved in SR-BI and ABCG1 mediated HDL endocytosis, is not yet proven. Instead, HDL transcytosis through endothelial cells might involve several redundant receptors since co-silencing of SR-BI and ABCG1 reduced HDL transcytosis by 30% only [93]. This strongly suggests involvement of other factors in HDL transcytosis. Given the high abundance of caveolae in endothelial cells, their contribution to HDL transcytosis seems plausible. However, a role of caveolae has so far only been shown in the transendothelial transport of LDL [95]. Interestingly, over-expression of endothelial caveolin-1 leads to enhanced atherosclerosis [96] which could be interpreted as a result of increased LDL transcytosis resulting in enhanced foam cell formation.

Glycosylphosphatidylinositol-anchored high density lipoprotein-binding protein 1 (GPIHBP1) was initially described as an HDL receptor and is highly expressed in endothelial cells in-vivo [97]. However, GPIHBP1 knockout mice have major defects in the lipolysis of triglyceride lipoproteins and not in HDL metabolism [98]. Moreover, GPIHBP-1 is not expressed in large artery endothelial cells, even though the large arteries display HDL transcytosis. This suggests that GPIHBP-1 does not mediate HDL transcytosis in vascular endothelial cells.

While vascular endothelial cells are studied extensively in terms of HDL transcytosis, HDL transport through lymphatic endothelial cells has been largely neglected previously. A recent report showed that lymphatic endothelial cells express ABCA1 and SR-BI, but not ABCG1 [92]. This study revealed that cholesterol removal from atherosclerotic plaques requires active transport of HDL through lymphatic endothelial cells, which is mediated by SR-BI.

### 5.4 Adipose tissue

Adipose tissue is the major storage site for triglycerides and thus plays a central role in energy metabolism. In addition, it is the largest reservoir for free cholesterol and a direct modulator of cholesterol metabolism: Adipocyte ABCA1 mediates cholesterol efflux in-vitro [99] and consistently, adipose tissue significantly contributes to HDL biogenesis in-vivo [100]. In addition, adipose tissue is involved in the clearance of HDL derived cholesterol from the circulation, which is mediated by SR-BI [101,102]. HDL endocytosis was described in adipocytes, but its significance is unclear. Like in other cell types, selective CE uptake exceeds holo-particle uptake in adipocytes [103,104]. Adipocytes express ecto-F_1_-ATPase and its expression increases during adipocyte differentiation [105]. Adipocyte ecto-F_1_-ATPase mediates endocytosis and resecretion of free apoA-I. However, this apoA-I recycling is not accompanied by cholesterol efflux and its physiological function is unknown yet [106]. If ecto-F_1_-ATPase is also involved in the regulation of HDL endocytosis remains to be investigated.

### 5.5 Platelets

Platelets are directly involved in atherosclerotic plaque formation and thrombosis. As HDL cholesterol levels are inversely related to thrombosis risk, the interaction of HDL with platelets is under extensive investigation [107]. Platelets express SR-BI and SR-BI knockout mice display defects in platelet structure and function [108]. However, this effect was largely attributed to dyslipidemia in these mice rather than to the absence of the receptor in platelets per se [109,110]. Evidence for a direct role of platelet SR-BI was provided recently in a study which identified HDL associated phospholipids as important modulators of the anti-thrombotic action of HDL [111]. Another study suggests that the reduction of platelet membrane cholesterol by efflux to reconstituted HDL can be utilized as a therapeutic approach to inhibit platelet activation [112]. However, the effects of HDL on platelet activation were generally attributed to alterations in cell signaling and a role for HDL endocytosis has not been investigated yet.

## 6 Conclusions and perspectives

Despite the fact that HDL endocytosis has been known for more than 40 years, its physiological relevance is still under debate. HDL endocytosis does not seem to be a major contributor to maintain cellular cholesterol homeostasis. It is generally accepted that selective lipid uptake exceeds HDL protein uptake and is highly efficient [5]. However, various pieces of evidence point towards a redundant function of HDL holo-particle uptake in cases where normal mechanisms to maintain cellular cholesterol homeostasis are disturbed.

In SR-BI knockout mice, HDL endocytosis in adrenals is enhanced, suggesting a compensation for reduced selective lipid uptake [72]. Further evidences for HDL endocytosis as a redundant pathway come from our findings in cells derived from patients with Niemann–Pick disease, where the LDL-receptor pathway and late endosomal cholesterol trafficking are impaired. In fibroblasts from these patients, HDL retro-endocytosis is still functional in maintaining cellular cholesterol homeostasis [113]. Similarly, HDL and LDL resecretion mediates cholesterol efflux in fibroblasts from patients with Tangier disease, where ABCA1 function and cholesterol efflux to apoA-I are defective (Neuhofer and Stangl, unpublished data).

In case of low cholesterol availability and high cholesterol demand, cells seem to adapt to HDL endocytosis and degradation to supply themselves with cholesterol [61]. Is HDL endocytosis therefore a redundant pathway that only becomes important in pathologic conditions? Importantly, redundancy is not only found in case of HDL pathways, but may also exist between lipoprotein classes, where one lipoprotein species steps in for another: In cells lacking a functional LDL-receptor, SR-BI mediates both selective and holo-particle uptake from LDL [29,114].

Another hypothesis for an essential function of holo-particle uptake is cholesterol delivery to distinct cellular pools. In hepatocytes, for instance, there is evidence for distinct functional cholesterol pools, one for bile-acid synthesis and one for direct cholesterol secretion into the bile [115]. One can speculate that HDL endocytosis transports cholesterol to an intracellular pool that is different from the one supplied by selective uptake.

Taken together, the role of HDL uptake and resecretion in cholesterol homeostasis is still insufficiently defined. However, the function of HDL as a transport vehicle is not restricted to cholesterol. Instead, HDL cargo comprises a multitude of lipid species, hormones, proteins and nucleic acids [3]. For instance, HDL can transport microRNAs to target cells to modulate gene expression [116]. Moreover, at least 50 distinct proteins are associated with HDL, many of which are related to anti-inflammatory and anti-oxidative functions [117,118]. It is interesting to hypothesize that HDL endocytosis has a yet elusive role in transfer of non-lipid cargo. HDL might acquire proteins and microRNAs during intracellular trafficking, and endocytosis in target cells might be required for unloading this non-lipid cargo.

## Figures and Tables

**Table 1:** HDL endocytosis in tissue culture.

Cell line	Tissue	Species	Ref.	Remark
HepG2	Liver	Human	[44,45,71,119,120]	
HepG2	Liver	Human	[49,50]	Polarized HepG2 cells
HUH-7	Liver	Human	⁎	
Primary hepatocytes	Liver	Mouse	[28]	Polarized murine hepatocytes
Primary hepatocytes	Liver	Mouse	[55,77]	
Hepa1-6	Liver	Mouse	⁎	
Non-parenchymal liver cells	Liver	Mouse	[79]	
FU5AH	Liver	Rat	[10]	
Aortic smooth muscle cells	Endothelium	Rat	[6]	1st report of HDL retro-endocytosis
Aortic endothelial cells	Endothelium	Rat	[121]	
Aortic endothelial cells	Endothelium	Bovine	[93,94]	
HUVEC	Endothelium	Human	⁎	
HCAEC	Endothelium	Human	⁎	
EPCs	Endothelium	Human	⁎	Endothelial progenitor cells
Peritoneal macrophages	Macrophage	Mouse	[8]	
Monocyte-derived macrophages	Macrophage	Human	[9]	
THP-1 monocyte/macrophage	Macrophage	Human	[29]	
Madin–Darby cells	Kidney	Canine	[28]	Polarized epithelial cells
HEK293	Kidney	Human	⁎	
Y1BS1	Adrenals	Human	[29]	
Caco-2	Intestine	Human	[46]	
3T3-L1 adipocytes	Adipocytes	Human	⁎	
Trophoblasts	Placenta	Human	[60]	
BeWO	Placenta	Human	[61]	
Prim. capillary endothelial cells	Brain	Porcine	[122]	In-vitro model for blood–brain-barrier
CHO	Ovaries	Hamster	[29,52,53,56]	
CHO7	Ovaries	Hamster	[61]	Degrade HDL efficiently
COS-7	Fibroblast	Monkey	[48]	

⁎ Röhrl, Fruhwürth, Srisen, Winter, Neumüller and Stangl, unpublished data.
